# Supplementary material for: Interspecific hierarchies from aggressiveness and body size among the invasive alien hornet, Vespa velutina nigrithorax, and five native hornets in South Korea
Source: PLoS One. 2020 Jul 28;15(7):e0226934. doi: 10.1371/journal.pone.0226934 (PMC7386586; doi:10.1371/journal.pone.0226934)
Supplement: S1 Table — (DOCX) [file pone.0226934.s001.docx]

S1 Table. Distribution of aggressiveness scores between an invasive alien hornet, *V. velutina*, and five native Korean hornet species

| Score | vel & sim | vel & man | vel & ana | vel & cra | vel & dyb |
| --- | --- | --- | --- | --- | --- |
| 2 : 0 | 73 | 3 | 10 | 13 | 7 |
| 3 : 0 | 31 | 1 | 2 | 3 | 1 |
| 4 : 0 | 4 | 0 | 0 | 0 | 0 |
| 1 : 1 | 10 | 5 | 4 | 9 | 4 |
| 0 : 2 | 28 | 39 | 39 | 49 | 73 |
| 0 : 3 | 7 | 42 | 12 | 19 | 42 |
| 0 : 4 | 0 | 11 | 0 | 0 | 5 |
| 0 : 5 | 0 | 3 | 0 | 0 | 0 |
| Total | 153 | 104 | 67 | 93 | 132 |
