# Supplementary material for: Interspecific hierarchies from aggressiveness and body size among the invasive alien hornet, Vespa velutina nigrithorax, and five native hornets in South Korea
Source: PLoS One. 2020 Jul 28;15(7):e0226934. doi: 10.1371/journal.pone.0226934 (PMC7386586; doi:10.1371/journal.pone.0226934)
Supplement: Attachment — Submitted filename: response to reviewers.docx [file pone.0226934.s004.docx]

**Question 1: All the P-values reported need to be associated to their statistical (e.g. t, F) and degrees of freedom (df). To report properly the statistical analyses all these features should be given in the text (or in tables in main text) together with the P value. Examples: t(28) = 2.6, p < .05 or F2,28= 55, P=0.01. These values should correspond to the t-tests or ANOVA global test, while the Tuckey a posteriori test should be given in the figures using asterisks or letters (I see this has been done in one of the figures), to show significant differences between different levels of a significant factor. In case of body size you report the F but not the df. Also this sentence should be slightly rewritten because the result of anova might give whether species (in general) differ in size, whereas it is the post hoc test that show which is different from which (which is not totally clear in the way it is written).**

**Response**: All the degrees of freedom mentioned by the reviewer were inserted.

The revised part is indicated in red letters.

Aggressive intensity, winning percentages, and aggressive behavior trends

In *V. velutina* vs. *V. simillima*, *V. velutina* was superior to *V. simillima* in 153 fights (t_(304)_=9.89, P < 0.001, Fig. 2A), and won 71% of the encounters (Fig. 3). *V. velutina* had a high rate of threatening behavior, such as rushing the opponent, and lifting the antennae and front legs and shaking the wings. *V. simillima*, however, rushed the opponent more often than *V. velutina* (Fig. 4A).

In *V. velutina* vs. *V. mandarinia*, *V. mandarinia* outperformed *V. velutina* in a total of 104 fights (t_(206)_=-22.75, P < 0.001, Fig. 2B) and won 91% of the encounters (Fig. 3). *V. mandarinia* displayed a high rate of grappling behavior, such as banging or pushing with the head (Fig. 4B). *V. mandarinia* also preyed upon *V. velutina*, although rarely.

In *V. velutina* vs. *V. analis*, *V. analis* was superior to *V. velutina* in 67 fights (t_(132)_=-8.38, P < 0.001, Fig. 2C) and won 76% of the encounters (Fig. 3). Concerning threatening behavior, *V. analis* displayed threat most often by rushing the opponent, and this species’ second favorite ploy was to fly threateningly over the adversary. In contrast, *V. velutina* made a weak show of rushing the opponent (Fig. 4C).

In contests between *V. velutina* and *V. crabro*, *V. crabro* was superior to *V. velutina* in 93 fights (t_(184)_=-9.62, P < 0.001, Fig. 2D) and won 73% of the encounters (Fig. 3). Lifting antennae and front legs and shaking wings were the most common threatening behaviors in *V. crabro,* and banging or pushing with the head were the most common grappling behaviors. In contrast, *V. velutina* displayed minor aggressive behavior such as threateningly flying over the opponent (Fig. 4D).

Finally, in encounters between *V. velutina* and *V. dybowskii,* *V. dybowskii* was superior to *V. velutina* in a total of 132 fights (t_(262)_=-24.22, P < 0.001, Fig. 2E) and won 91% of the contests (Fig. 3). The most common threatening behavior employed by *V. dybowskii* was chasing the opponent, and the second most common action was rushing the opponent; chasing and grabbing were the most common grappling behaviors for this species. *V. velutina*’s counterattack action was to fly threateningly over the opponent but not vigorously or often (Fig. 4E).

Fig. 2. Aggressiveness scores between an invasive alien hornet, *V. velutina*, and five native Korean hornet species. Scores are displayed as mean values ± SD. A: *V. velutina* (score 1.73±1.11) vs *Vespa simillima* (score 0.57±0.94), t_(304)_=9.89, P<0.001; B: *V. velutina* (score 0.13±0.48) vs *Vespa mandarinia* (score 2.58±0.98), t_(206)_=-22.75, P<0.001; C: *V. velutina* (score 0.45±0.86) vs *Vespa analis* (score 1.76±0.96), t_(132)_=-8.38, P<0.001; D: *V. velutina* (score 0.47±0.86) vs *Vespa crabro* (score 1.76±0.97), t_(184)_=-9.62, P<0.001; E: *V. velutina* (score 0.16±0.54) vs *Vespa dybowskii* (score 2.24±0.83), t_(262)_=-24.22, P<0.001. (See S1 table).

Body size

The body size of *V. velutina* was 8.04 ± 0.41 mm, which was slightly larger than *V. simillima* 7.80 ± 0.29 mm. *V. dybowskii* was 8.88 ± 0.49 mm, *V. analis* was 10.14 ± 0.51 mm, *V. crabro* was 9.82 ± 0.4 mm, and *V. mandarinia* was 13.21 ± 0.83 mm. Thus, except for *V. simillima*, all species were larger than *V. velutina*. There were significant differences in body size among *Vespa* species (F_(5, 174)_ = 434.9, P <0.001), being the differences significant between all the species except for *V. simillima* and *V. velutina*, and *V. analis* and *V. crabro* (Fig. 5).

Fig. 5. Body size differences between an invasive alien hornet, *V. velutina* and five native Korean hornet species. (F_(5,174)_=434.9, P<0.001).

**Question 2:** Regarding the figures, it is not necessary to write in each panel ‘mean ±SD’. Instead, say it in the legend, and show their significance (using for instance asterisk that are mentioned in the legend).

**Response:** Delete ‘mean ±SD’ in the figure and indicate significance in alphabet

**Question 3:** Check also for small typological mistakes that appear along the text.

**Response:** Some modifications were made to the italics and notation of references.
